# Sustainable canal lining with reinforced composite concrete and geo-textile

**DOI:** 10.1016/j.mex.2025.103704

**Published:** 2025-11-02

**Authors:** Parul Gajbhiye, Nyla Vaidya, Hamza Erfan, Dhrumit Jain, Mategaonkar Meenal

**Affiliations:** Mukesh Patel School of Technology Management & Engineering, SVKM's NMIMS, Mumbai, India

**Keywords:** Canal lining, Sustainable solutions, Interlocking composite concrete plates

## Abstract

Conventional canal linings are prone to seepage, erosion, and structural deterioration, resulting in water losses and depletion of natural resources. Addressing these challenges is essential for improving irrigation efficiency and ensuring sustainable water management. This study presents a novel canal lining method that combines composite geotextile with interlocking composite concrete plates reinforced by steel bars. The production process begins with the placement of a composite geotextile fabric, serving as a filtration and stabilization layer to enhance erosion control and load distribution. Prefabricated concrete plates, designed with admixtures to increase strength and durability, are then interlocked and reinforced with steel bars to provide additional crack resistance and structural stability. This integrated system simplifies installation, reduces construction time, and forms a durable, impermeable lining capable of mitigating seepage, structural damage, and sedimentation in irrigation canals. Laboratory investigations demonstrate a 12% increase in crushing strength and a 30% improvement in load-carrying capacity, confirming the reliability and sustainability of the proposed canal lining solution. This study introduces a production method for canal lining that integrates interlocking composite concrete plates, reinforced with bars and composite geotextile, designed to mitigate seepage, structural damage, and sedimentation while improving load distribution and durability.

• The lining system is produced by casting concrete with selected admixtures and embedding geotextile layers, resulting in laboratory-tested improvements of 12 % in crushing strength and 30 % in load-carrying capacity, ensuring enhanced structural reliability.
• The developed method not only extends the service life of irrigation canals and reduces water losses but also supports environmentally sustainable water management practices with practical applications in agriculture and resource conservation.

## Graphical abstract

**Figure:**
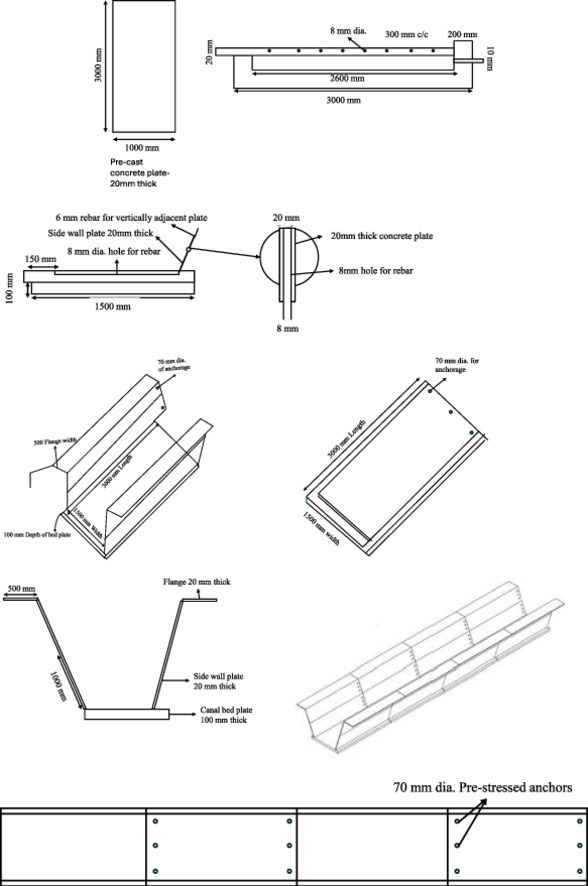

Specifications table

**Table:** 

Subject area	Engineering
More specific subject area	*Water Resource Engineering*
Name of your method	*Interlocking composite concrete plates for canal lining*
Name and reference of original method	Central Water Commission [3]. Evaluation of Canal Infrastructure and Structural Failures. Jadhav, P., Thokal.R., Mane.M., Bhange, H. and Kale.S [5]. Improving Conveyance Efficiency through Canal Lining in Command Area: A Case Study*.* International Journal of Engineering Innovation & Research Volume 3, Issue 6. Yao,L., Shaoyuan, F., Xiaomin, M., Zailin H., Shaozhong K., D.A. Barry [16],Coupled effects of canal lining and multi-layered soil structure on canal seepage and soil water dynamics, Journal of Hydrology, Volumes 430–431,Pages 91–102,
Resource availability	*If applicable, include links to the resources necessary to reproduce your method (e.g., equipment, data, software, hardware, reagents).*

## Background

Irrigation infrastructure in India, particularly in rural regions, has long faced neglect, inefficiency, and inadequate maintenance, creating significant challenges in meeting the increasing water demands of a rapidly growing population [11]. Canal systems, essential for distributing water to agricultural lands, are especially vulnerable due to aging structures and outdated lining materials. Poorly constructed or maintained linings result in substantial water losses, reduced irrigation efficiency, and lower crop productivity [4]. In water-stressed states such as Maharashtra, seepage from inefficient canals degrades soil quality, contributes to groundwater contamination, and exacerbates water scarcity [2]. Structural issues including cracking, sedimentation, and erosion further impair operational efficiency, leading to inconsistent water delivery and disproportionately affecting small and marginal farmers who rely heavily on irrigation [12,15].

Traditional canal lining materials, such as concrete and compacted earth, are prone to long-term degradation and require frequent repairs, increasing operational and maintenance costs [10]. Properly constructed linings can significantly reduce water losses—up to 78 % in certain systems—but may also affect groundwater recharge, highlighting the need for balanced design solutions [9]. Despite their critical role, canal systems are often repaired reactively, with limited funding resulting in temporary fixes rather than sustainable, long-term solutions [5,14]. Substandard construction practices and low-quality materials further accelerate deterioration, reinforcing the cycle of inefficiency [7,16].

In Maharashtra, inefficient canal linings can result in the loss of up to 40 % of conveyed water, reducing availability for irrigation and other uses [6]. Structural failures such as cracking, seepage, and sediment accumulation not only increase maintenance costs but also compromise water delivery efficiency and threaten agricultural productivity [3,8]. Financial constraints often limit authorities to temporary fixes, transferring the burden to farmers who face higher production costs and reduced yields [1]. These challenges underline the urgent need for durable and sustainable canal lining solutions that improve water retention, minimize seepage, and enhance long-term structural performance.

Addressing these challenges demands a shift toward innovative and durable solutions, such as advanced waterproofing materials and sustainable canal lining designs is proposed in this study. Such measures can reduce seepage, enhance structural integrity, and ensure efficient water delivery, alleviating the financial and environmental pressures on irrigation infrastructure while supporting agricultural productivity.

## Method details

### Proposed design

In conventional canal lining structures, common failure modes include cracking due to tensile stresses, erosion and washout at joints, seepage through poorly compacted soil or damaged lining, settlement of the canal bed, and root or vegetation penetration that compromises structural integrity. These failures often lead to water loss, reduced hydraulic efficiency, and frequent maintenance requirements.

**Fig. 1:**
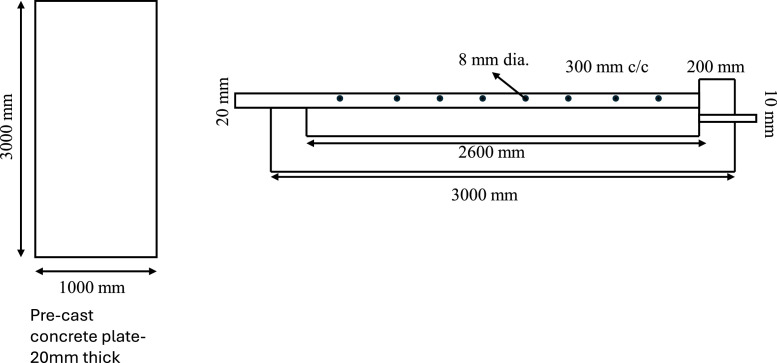
Schematic diagram of the interlocking plates (Single panel detailing).

The proposed sustainable waterproofing solution for canal linings leverages an innovative interlocking technology utilizing custom-designed concrete plates to ensure enhanced durability and water resistance. Each plate measures 3000 mm x 1000 mm x 100 mm providing substantial coverage while maintaining manageable dimensions for transportation and installation (Fig. 1). The plates are equipped with collars at both ends, measuring 200 mm x150 mm x 50 mm in thickness, which enable secure lap joint connections between consecutive plates.

**Fig. 2:**
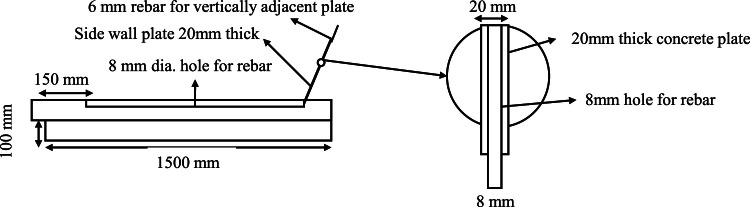
Plates with holes for reinforcement.

To reinforce structural integrity, each plate is embedded with 8 mm diameter holes, drilled at intervals of 300 mm center-to-center, allowing for reinforcement bars that bond the side-wall plates to form a unified, homogeneous structure (Fig. 2).

These collars run along the length of each plate, facilitating a robust lap joint mechanism with a 200 mm overlap. This joint is anchored into the ground using a prestressed anchorage system, enhancing the stability of the canal lining. Additionally, the incorporation of a bi-axial geogrid during the casting process further optimizes the plates for improved load-bearing capacity, ease of handling, and uniform stress distribution, ultimately contributing to a reliable and sustainable waterproofing solution for canal infrastructures.

### Interlocking technology of plates in side-walls

**Fig. 3:**
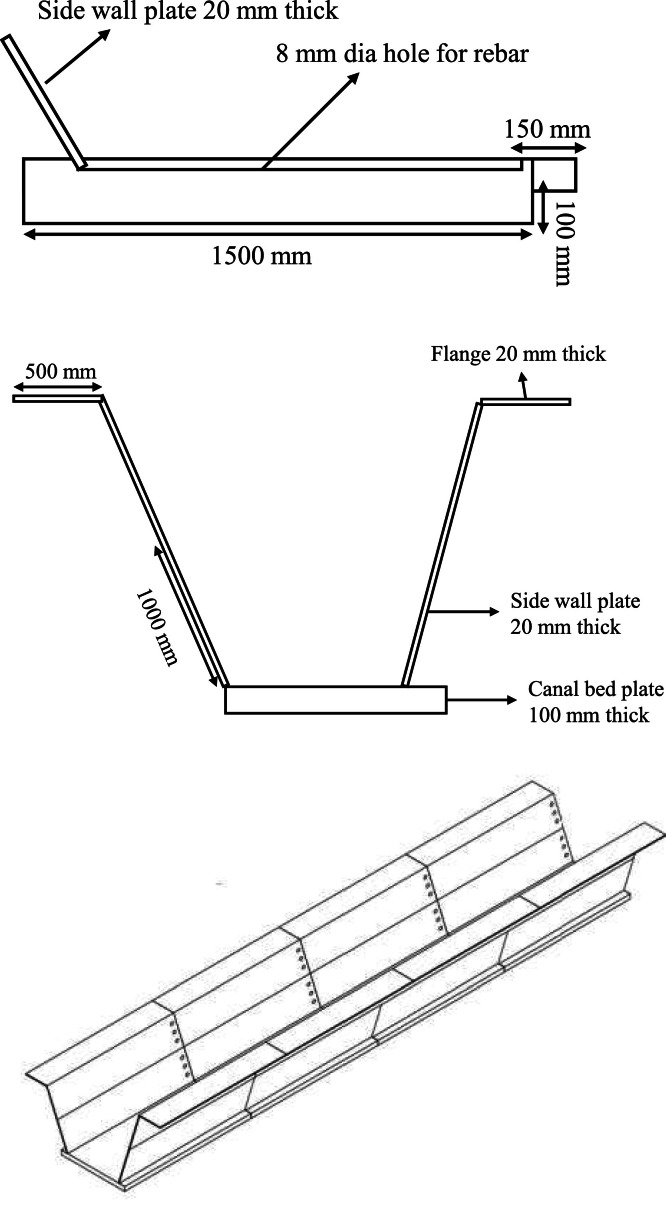
Interlocking in side wall plate.

Each side wall plate is precisely dimensioned at 3000 mm x 1000 mm x 20 mm, with collars measuring 200 mm x 100 mm x 10 mm integrated along the plate’s longitudinal edges to enable a secure 200 mm lap joint between consecutive plates (Fig.3a & 3b).

**Fig. 4:**
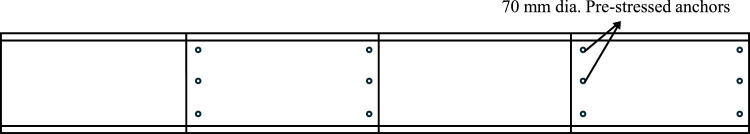
Anchorage system.

This lap joint is anchored into the ground via a prestressed anchorage mechanism, enhancing lateral stability under hydrostatic pressure. For a unified load- bearing structure, each plate is perforated with 8 mm diameter holes at 300 mm center-to-center, allowing reinforcement bars to pass through and interconnect with the canal bed, forming a continuous reinforced matrix across the side walls and bed. Additionally, 6 mm reinforcement bars project from the bed plates in precise alignment with the open canal perimeter, providing controlled angular alignment to achieve the desired slope for the side walls and effectively distribute both vertical and lateral loads to reduce stress concentrations and enhance structural resilience (Fig. 4).

**Fig. 5:**
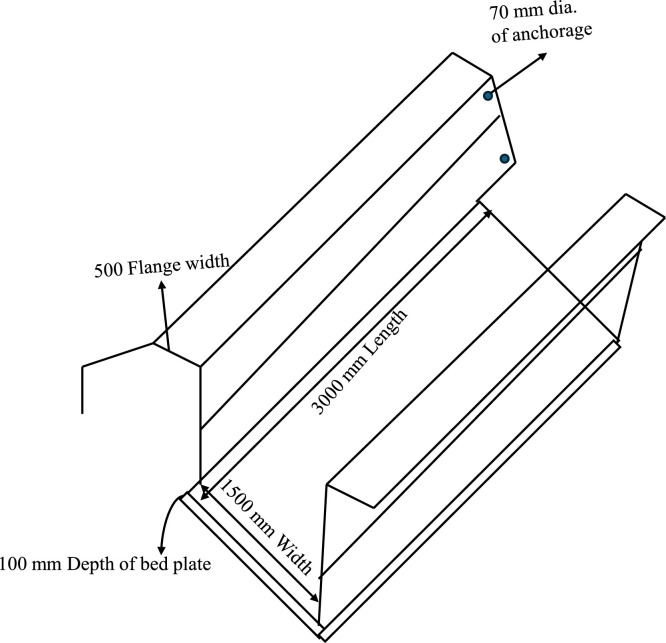
Details of the canal lining.

**Fig. 6:**
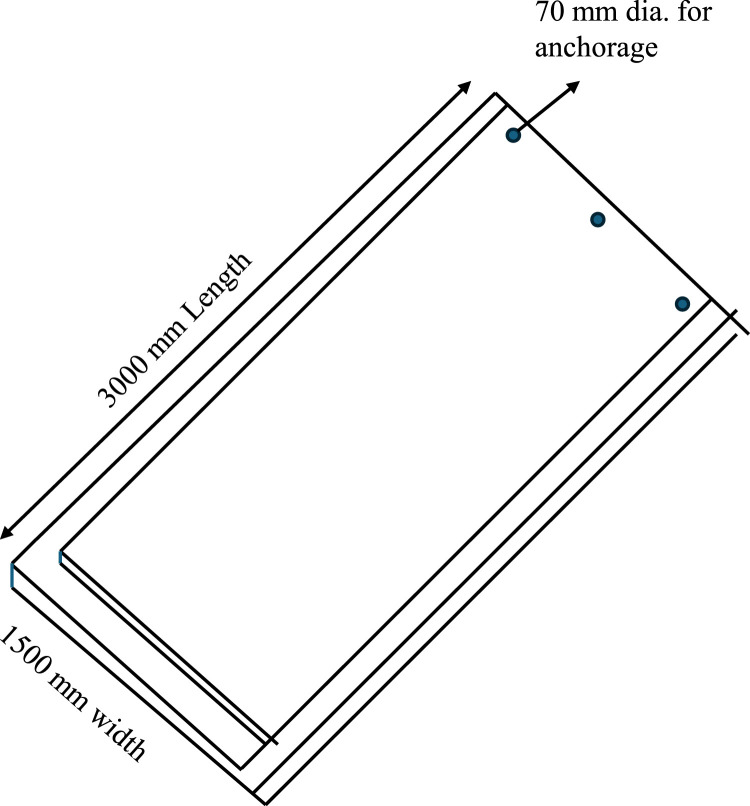
Detailed section of the bed plate.

To prevent leakage, the vertically stacked plates incorporate a rubber-based sealant at each interface, creating a flexible yet impermeable seal that accommodates minor movements without compromising watertightness. The grouting of the 8 mm perforations further serves as an in-situ sealant, reducing leakage pathways and reinforcing the structural bond between plates. This integrated system, combining mechanical interlocks and advanced sealant applications, ensures that the side wall lining is both durable and sustainable, reducing water loss and maintenance needs over the canal’s operational life. The detailed section and bed lining structure is given in Fig.5& Fig.6.

### Ground improvement technology

The methodology of this study involves designing and implementing canal lining using composite concrete in combination with a geotextile layer, tailored to the soil conditions of the study area. The primary soils are clayey and silty, with moderate compressibility and variable moisture retention, which can lead to permeability issues and instability under hydraulic loading [13]. To address these challenges, a high density polyethylene (HDPE) geotextile is first laid as a filtration and stabilization layer between the native soil and the canal lining. This geotextile blocks water leakage, reduces seepage, prevents soil erosion, minimizes risks of settlement, and inhibits root penetration or vegetation growth that could compromise the lining. Prefabricated interlocking composite concrete plates, reinforced with steel bars and optimized with admixtures for compressive strength and durability, are then installed over the geotextile.

**Fig. 7:**
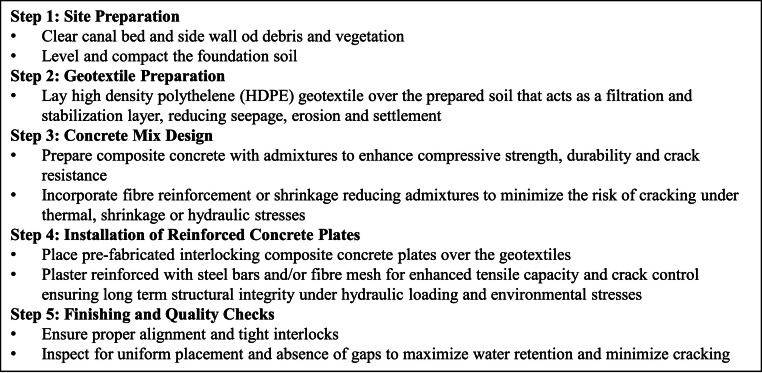
Step by Step procedure for laying of canal lining Two mix designs are proposed for canal lining with SBR latex admixture and one without admixtures (Table 1).

The interlocking design enhances structural integrity, minimizes cracking, and facilitates water retention, creating a durable, impermeable, and sustainable canal lining system that improves both hydraulic and structural performance. Rubber-based sealants and grouted perforations further improve watertightness and flexibility. Fig. 7 gives the step by step procedure for laying of canal lining using geotextile and composite concrete.

## Method validation

### Results and discussion

To see the durability and strength of the concrete lining, the crushing strength tests are conducted on the concrete cubes, both with and without admixture. It includes the crushing strength in N/mm² and the corresponding maximum load-carrying capacity in kN for each cube type. The data demonstrates the strength development of both standard RCC M30 and RCC M30 with admixture over time.Table 2 summarises the crushing strength after 7th,14th and 28 days.

**Table 1:** Proposed mix designs.

Material (kg)	Mix design	
	Without admixtures	With admixtures
Cement	413.716	435.00
Course aggregate	1226.360	1135.64
Fine aggregate	599.970	555.59
Water	186.173	186.173
Admixture	—	43.5
Ratio	1:2.96:1.45:0.45	1:2.61:1.28:0.1:0.45

**Table 2:** Crushing strength of the concrete to be used for the canal lining.

Cube No.	Type of Cube	Age (days)	Crushing strength (N/mm2)	Max. load carrying capacity (kN)
1	RCC M30	7	17.8	400
2	RCC M30	14	23	540
3	RCC M30	28	30.9	700
4	RCC M30 with Admixture	7	22.07	410
5	RCC M30 with Admixture	14	31.16	600
6	RCC M30 with Admixture	28	40.25	790

The crushing strength and load-carrying capacity tests for RCC M30 and RCC M30 with SBR latex admixture, outlined in Table 2, highlight the effects of admixtures on compressive strength and early strength development. For RCC M30 without admixture, a slump of 75 mm ensures good workability suitable for prefabrication. As per IS 456:2000, M30 grade concrete aims for a compressive strength of 30 N/mm^2^at 28 days [BIS, 2000]. The 7-day strength reached 17.8 N/mm^2^, close to the theoretical range of 18–20 N/mm\u00b2, with a 14-day strength of 23 N/mm\u00b2 matching expected values (22–24 N/mm^2^). By 28 days, the strength exceeded expectations at 30.9 N/mm^2^, indicating effective curing and mix proportioning. For RCC M30 with SBR latex, the 7-day strength improved to 22.07 N/mm^2^, surpassing the range for plain concrete and demonstrating the admixture's role in enhancing early strength. At 14 days, strength rose to 31.16 N/mm^2^, exceeding the 28-day theoretical target, attributed to improved bonding and matrix density. Load-carrying capacity showed a similar trend, increasing from 400 kN to 700 kN for plain RCC and achieving higher early-stage values with admixture. The inclusion of SBR latex significantly enhanced early compressive strength and impermeability, making it ideal for canal lining systems.

Conventional canal linings often emphasize compressive strength while neglecting cracking behavior, which critically affects durability and service life. Cracks arising from shrinkage, thermal stresses, or cyclic loading can lead to seepage, erosion, and structural deterioration. The proposed canal lining system addresses these issues by combining reinforced interlocking composite concrete plates with a geotextile layer, which redistributes stresses, reduces localized cracking, and enhances structural reliability. Compared to traditional linings, the integrated system improves load distribution, water retention, and erosion control while simplifying installation and allowing replacement of individual units, resulting in a more durable and cost-effective solution.

Steel corrosion under long-term water exposure is mitigated by embedding reinforcement within high-quality concrete, covering it with geotextile, and using corrosion-resistant admixtures. Laboratory tests confirm increased compressive strength and load capacity, demonstrating that the proposed method not only enhances structural performance but also ensures long-term sustainability and reliability of canal infrastructure.

While initial costs are higher, reduced maintenance offers long-term economic benefits, as indicated in the cost comparison in Table 3. This supports the use of advanced admixtures for sustainable infrastructure.

**Table 3:** Cost analysis of the traditional and proposed method of canal lining.

Parameter	Traditional Canal Lining	Proposed Canal Lining
Initial Cost	Moderate	High
Maintenance (10 years)	High	Low
Total Cost (10 years)	Very High	Moderate

## Conclusion

Water seepage and leakage through canal linings are major obstacles to effective water management, with traditional methods like thin cement coatings often failing due to wear and difficulty in replacement. This study proposes prefabricated interlocking concrete plates as a sustainable solution, offering easier installation, reduced maintenance, and superior performance. Tests show a 30 % increase in concrete’s crushing strength and a 12 % improvement in load-carrying capacity when enhanced with admixtures. Validated through 2D and 3D modeling, the design ensures structural stability and efficiency. The system minimizes seepage, water loss, and structural damage risks while incorporating geo-textiles to control erosion and sedimentation. It aligns with Sustainable Development Goals, particularly SDG 6 (clean water and sanitation) by conserving water and improving irrigation, and SDG 9 (industry, innovation, and infrastructure) by advancing resilient infrastructure with innovative materials. This approach offers a transformative pathway for sustainable water resource management.

## Limitations

This study is limited by high initial costs, untested long-term field performance, and potential maintenance needs for geo-textiles. Scalability challenges in rural areas and environmental impacts of concrete production also require further evaluation. Broader adoption depends on policy support, infrastructure readiness, and integration into sustainable water management systems.

## CRediT author statement

Parul: Conceptualization, Methodology, Software Nyla, Hamza: Writing- Original draft preparation. Dhrumit: Data Curation, Investigation. *Meenal:* Supervision, Writing- Reviewing and Editing

## Supplementary material *and/or* additional information [OPTIONAL]

None

## Declaration of interests

The authors declare that they have no known competing financial interests or personal relationships that could have appeared to influence the work reported in this paper.

## Data Availability

No data was used for the research described in the article.
